# What is the Association Between Economic Growth and Health Equity? A Cross-National Study of 83 Low- and Middle-income Countries

**DOI:** 10.1177/27551938251345969

**Published:** 2025-05-29

**Authors:** Toby Freeman, Hailay Abrha Gesesew, Clare Bambra, Heather Brown, Shahid Ullah, Jennie Popay, Fran Baum

**Affiliations:** 1 Stretton Health Equity, 1066 University of Adelaide, Adelaide, Australia; 2 Research Centre for Public Health, Equity and Human Flourishing, 386703 Torrens University Australia, Adelaide, Australia; 3 247536 Mekelle University College of Health Sciences, Mekelle, Ethiopia; 4 Population Health Sciences Institute, Newcastle University Faculty of Medical Sciences, Newcastle upon Tyne, UK; 5 4396 Lancaster University, Lancaster, UK; 6 College of Medicine and Public Health, Flinders University, Adelaide, Australia; 7 Division of Health Research, Faculty of Health & Medicine, 4396 Lancaster University, Lancaster, UK

**Keywords:** health inequities, mortality, global health, socioeconomic status, gross domestic product

## Abstract

Inequities in infant and child mortality are an urgent public health issue for lower- and middle-income countries (LMICs). We sought to establish whether gross domestic product (GDP) is associated with the extent of health inequalities within LMICs. We conducted a secondary analysis of publicly available health equity data from the Health Equity Database of LMICs and GDP data from the World Bank. We used infant and under-five mortality rates by socioeconomic quintile. The slope of inequality index and relative index of inequality were calculated for both outcomes for each country (*n* = 83). Mixed effect linear regression analysis was used to investigate the relationship with GDP. Higher GDP was associated with a significant decrease in absolute socioeconomic inequities in both infant (*f*^2^ = 0.26) and under-five mortality (*f*^2^ = 0.33). Higher GDP was also associated with relative inequities in infant (*f*^2^ = 0.08) and under-five mortality (*f*^2^ = 0.07). Thus, increasing GDP may help reduce absolute inequities in infant and under-five mortality, but may increase relative inequities. Understanding drivers of the distribution of wealth and income to flatten the socioeconomic gradient in health are crucial to reducing health inequities.

Health inequities are systematic inequalities in health caused by unfair distribution of resources or other unjust processes, including unjust distribution of income and wealth, and factors such as racism, and other discrimination.^1^ Health equity is a global concern. Health inequities exist *between* countries and *within* countries. In terms of inequities between countries, life expectancy is a commonly used indicator of a population's health. There are large global inequities in life expectancy, ranging from Chad with a life expectancy of 52.8 to Hong Kong with a life expectancy of 85.4^2^—a span of more than 30 years’ difference driven by inequities in social determinants of health.^3^

A country's life expectancy has long been associated with national income as measured by gross domestic product (GDP); the greater a nation's wealth, in general, the longer its life expectancy. Gains in life expectancy associated with increased GDP particularly occur amongst low- and middle-income countries (LMICs), with life expectancy benefits of a higher GDP subsequently tapering off among richer countries.^4,5^ This relationship was demonstrated by Samuel Preston in 1975 and is known as the Preston Curve.^4,5^ The research on GDP and life expectancy shows that LMICs have lower life expectancies than higher income countries. Childhood mortality is also higher in LMICs than high-income countries, including infant mortality and under-five mortality, and this gap is growing.^6^ Excess deaths since the onset of the COVID-19 pandemic has been estimated to increase child mortality in LMICs by 3.6 percent.^7^ One of the Millenium Development Goals was to reduce child mortality by two thirds,^8^ and this focus remains in the Sustainable Development Goals, with target 3.2 aiming for reductions in neonatal and under-five mortality.^9^

While we understand some of the factors that may influence a nation's life expectancy in relation to its wealth,^10,11^ there is less research on what factors drive health inequities within countries—that is, the *equitable distribution* of a nation's life expectancy, mortality, or other health outcomes.^12^ This case is particularly evident in LMICs,^13,14^ made all the more urgent because most LMICs are failing to reduce within-country health inequities,^15^ while health inequities are growing in high-income countries including Australia,^16–18^ North America, the United Kingdom, and Europe,^19^ even before the COVID-19 pandemic further exacerbated health inequities globally.^20–24^

Jetter and colleagues wrote of the Preston Curve, “the predominant medicine for longer lives seems to be raising the level of income per capita” (5, p. 1388), and this emphasis on increasing GDP persists as evidenced by target 8.1 in the Sustainable Development Goals of “at least 7 per cent gross domestic product growth per annum in the least developed countries”.^9^ We seek to understand if raising GDP is also the predominant medicine for decreasing health inequities in LMICs, or whether health inequities tend to persist even at higher levels of GDP. Findings for health equity may be different from overall health outcomes because health equity is sensitive to the distribution of social determinants of health, such as income, employment, and housing, within a population, as well as social inclusion and exclusion processes such as racism, gender inequities, and other discrimination.^25^ Thus, as a first step to understanding the drivers of health inequities, our research question was the following: Is there an association between national income (GDP) and the extent of health inequities within a country among low- and middle-income countries?

Some measures of health are comparable between countries, with life expectancy readily available for almost all countries. Health inequities are more difficult to compare between countries. There are two main approaches to measuring health inequities within a country in a way that allows comparisons. The first option is to calculate a single measure that captures variation in health among individuals in the population, such as a concentration index—much as is often done to measure the extent of income and wealth inequities.^26^ These measures capture the extent of variation in health but not socioeconomic, racial, or other inequities in health.

The second option is a bivariate approach, which looks at the distribution of health along socioeconomic or other inequality lines—for example, examining the relationship between health outcomes and income, level of education, or by geography or race. This analysis generates measures of the extent of inequities such as the slope index of inequality (SII) or relative index of inequality (RII), which measure the social gradient of health within a country. To conduct such bivariate examinations of health inequities between countries, however, requires both health outcome measures and socioeconomic or other inequalities measures that are available and comparable across countries.^27^

Beckfield and Krieger^28^ in their review found few studies that compared health inequities between countries and noted that 84 percent of the studies they found came from high-income countries. Only a handful more studies comparing health inequities between countries are evident since their review.^29–32^ Such studies have typically relied on survey data for health and/or socioeconomic measures.^29–31,33^ Some studies complement this with register-based mortality data.^29,30^ For bivariate approaches to measuring health inequities, there have been a range of socioeconomic measures selected, including education,^29^ income, and wealth.^31,33^ This body of work has tracked changes in health inequities over time,^34–36^ and there has been a particular strand of research that has investigated the effect of different welfare regimes^12,19,28^ that has yielded important findings, but also found puzzles, such as the strongest welfare states not having the lowest health inequities as would be expected, that challenge the ease of generating clear findings on inequities.^12^

Three studies were found that had examined the relationship between GDP and health inequities. Eozenou and colleagues^15^ found the concentration indices for under-five mortality and stunting were both strongly correlated with real GDP per capita, using data from 91 LMICs for under-five mortality and from 102 LMICs for stunting. This article adds to their article consideration of SIIs and RIIs to examine absolute and relative inequities, and inclusion of infant mortality measures. Baker and colleagues^32^ calculated the SII and RII for infant mortality for 48 LMICs using longitudinal panel data and found no evidence of a relationship between infant mortality inequities and GDP, though they did find government expenditure, especially expenditure in non-health sectors, was associated with lower infant mortality inequities. This research adds to this study updated data, inclusion of under-five mortality as well as infant mortality, and a larger dataset of 83 LMICs. Costa-Font and Cowell, using a dataset of 70 LMICs, showed that the relationship between GDP and health inequities varied according to the approach taken to calculating inequities in self-assessed health—varying between a positive relationship, negative relationship, or no relationship.^37^ This research adds to that study by repeating the examination using mortality data rather than self-assessed health.

## Methods

### Health Equity Data

The most comprehensive data source for comparable within-nation health equity indicators is the World Health Organization's Health Equity Monitor dataset.^38^ The dataset includes infant mortality rates and under-five mortality rates for 92 LMICs calculated from the internationally standardised Demographic and Health Surveys (and for under-five mortality, Multiple Indicator Cluster Surveys and Reproductive Health Surveys) conducted in LMICs.^38^ Infant and under-five mortality are crucial health indicators for LMICs because infant and under-five mortality rates in LMICs are seven and eight times as high, respectively, as in high-income countries.^39^ GDP has been found to be negatively associated with infant and child mortality rates.^40^ But there are differences in the determinants of infant and under-five mortality. For example, Memon and colleagues^41^ found household wealth predicted neonatal mortality but not under-five mortality, while distance to a health facility predicted only under-five mortality. Morakinyo and Fagbamigbe noted differences in countries’ longitudinal patterns of infant and under-five mortality, suggesting they are affected by different factors.^42^ Thus, we include both measures of mortality to ensure the influence of GDP on inequities is more comprehensively captured in our analysis. The infant and under-five mortality rates in the Health Equity Monitor dataset are expressed as deaths per 1,000 live births, and are available by wealth quintile. These quintiles are derived from a household wealth index, descibed by Health Equity Monitor as “country-specific indices … based on owning selected assets and having access to certain services and constructed using principal component analysis”.^38^ The health equity data for infant and under-five mortality were available for different time points per country. For 83 of the 92 countries, the most recent time point was between 2010 and 2019. Thus, the nine countries with a latest time point older than 2010 were excluded from analysis on the grounds that the age of the data made them less comparable. In total, 287 data points were recorded for 83 countries, each with a varying number of years of data.

### Gross Domestic Product Data

GDP per capita data were retrieved from the World Bank.^2^ We used GDP per capita converted to constant 2011 international dollars, which adjusts for purchasing power parity, to allow for meaningful comparison between countries. GDP was retrieved for the year matching the year of the most recent health equity data we had available from the Health Equity Monitor and other sources (i.e., if the health data was for 2010, we used GDP data from 2010). GDP was available for all 83 included countries.

## Analysis

We calculated the slope index of inequality (SII, measuring absolute inequities) and relative index of inequality (RII, measuring relative inequities) for infant and under-five mortality rates for each country (*n* = 83). These measures were chosen as they use information from across the socioeconomic gradient, rather than only the top and bottom quintiles.^43–45^ Both were calculated because researchers have argued that examining both absolute and relative inequities are important.^29,45,46^ Absolute inequities capture the raw difference between rates across the socioeconomic gradient (e.g., the gap in life expectancy between the rich and the poor expressed in years; in Australia, there is a 2.6 year gap in life expectancy between the richest and poorest areas^47^), while relative inequities capture this difference as a proportion of a referent rate (e.g., a rate ratio of mortality comparing the richest and poorest quintiles; in Australia, the rate ratio for premature mortality is 1.98, meaning premature mortality is 1.98 times higher in the poorest areas compared to the richest areas^18,48^) Absolute and relative inequities are affected by the overall rate differently—as mortality rates decrease, relative inequities are likely to increase, while absolute inequities are likely to decrease.^46,48^ Thus, including both gives a fuller picture of the relationship between GDP and health inequities.

The indexes represent outcomes regressed by quintile rank, to provide an estimation of the extent of inequities across the socioeconomic gradient.^32^ Like a regression coefficient, the further the indexes are from zero, the greater the socioeconomic inequities in the outcome.

We followed the method for SIIs and RIIs outlined in McKinnon and colleagues.^45^ For each country, SIIs and RIIs were calculated from the socioeconomic quintile specific infant and under-five mortality rates. We linearly regressed the quintile mortality rates on quintile rank to calculate the SII for each country, then divided the SII by the mean mortality rate for the country to calculate the RII, rather than the approach sometimes used where RII = *h*(1)/*h*(0).^44^

To investigate the relationship between GDP and health equity, four mixed effect linear regressions models were conducted on the SII and RII for infant and under-five mortality rates due to the hierarchical structure of the data. The hierarchy of the data was based on multiple years nested with each country. Countries were treated as random effects, and main effects were log GDP due to the non-linear nature of GDP. Standard scatter diagrams were used to visualize the relationships between health inequity measures and log GDP. Adjusted R-square was calculated for model goodness of fit and Cohen's *f*^2^ effect size^49^ for the magnitude of the relationship between log GDP and the index of inequality. Statistical analysis was performed using Stata version 16.1 (StataCorp, Texas, USA) and R software version 4.4.2.

As all analyses were conducted on publicly available data sets that used aggregated data, no ethics approvals were required.

## Results

The 83 countries spanned low-income (*n* = 24), lower middle-income (*n* = 38), and upper middle-income countries (*n* = 21). They included countries in the World Bank regions of Sub-Saharan Africa (*n* = 41), Latin America and the Caribbean (*n* = 11), Europe and Central Asia (*n* = 8), East Asia and the Pacific (*n* = 11), the Middle East and North Africa (*n* = 6), and South Asia (*n* = 6).

The SII and RII for the most recent year for each included country for infant mortality are shown in Table 1, and for under-five mortality in Table 2, along with the mortality rates for the richest and poorest quintiles. In some countries, health inequities were very large—for example, in Nigeria in 2018, the least wealthy quartile had almost twice the rate of infant mortality (78.1 [71.6-84.6]) compared to the wealthiest quintile (39.6 [34.1-45.2]), representing an extra 38 infants dying for every 1,000 live births in the poorest quintile. These inequities were evident at all levels of national income. As an example, Türkiye (Turkey) had the highest GDP in our sample, yet in 2013 Türkiye's infant mortality rate in the lowest wealth quintile (22.6 [15.3-30.0] was almost three times as high as the infant mortality rate in the wealthiest quintile (7.8 [0.0-16.3]).

**Table 1.** Slope Index of Inequality (SII) and Relative Index of Inequality (RII) (Higher = More Unequal) for Infant Mortality Rate (IMR; Deaths per 1,000 Live Births) for the Most Recent Year for Each Country Included.

Year	Country	SII	RII	IMR for Wealth Quintile 5 (richest) – Quintile 1 (poorest) with 95% CIs
	*Average*	*18*.*3*	*0*.*44*	*Q5: 29.0 Q1: 45.5*
	*Range*	*0.9-55.4*	*0.01-1.57*	*Q5: 0.4-78.6 Q1: 11.1-88.6*
2015	Afghanistan	27.3	0.55	34.5 [27.1-41.9]-61.8 [53.8-69.8]
2017	Albania	11.6	1.00	0.4 [0.0-1.1]-12.0 [3.9-20.2]
2018	Algeria	7.4	0.39	12.8 [6.7-18.9]-20.2 [16.0-24.5]
2015	Angola	37.9	0.79	24.6 [15.4-33.8]-62.4 [52.7-72.2]
2015	Armenia	8.1	0.34	4.5 [0.0-10.1]-12.6 [4.2-21.1]
2019	Bangladesh	17.8	0.28	24.0 [20.0-28.0]-41.9 [37.4-46.4]
2015	Belize	12.6	1.02	3.2 [0.0-7.2]-15.8 [5.6-26.0]
2017	Benin	23.9	0.33	39.3 [31.4-47.3]-63.2 [54.4-72.0]
2010	Burkina Faso	35.1	0.38	53.5 [43.9-63.2]-88.6 [79.8-97.4]
2016	Burundi	30.7	0.48	35.8 [25.2-46.5]-66.5 [58.4-74.7]
2014	Cambodia	46.7	0.71	15.6 [10.0-21.1]-62.3 [49.0-75.5]
2018	Cameroon	24.3	0.36	38.2 [30.5-45.9]-62.6 [50.9-74.2]
2018	Central African Republic	37.8	0.46	45.1 [36.0-54.2]-82.9 [69.4-96.4]
2019	Chad	1.0	0.01	62.7 [52.7-72.8]-63.8 [56.0-71.5]
2015	Colombia	16.5	0.78	5.4 [0.9-9.8]-21.8 [16.8-26.8]
2012	Comoros	2.7	0.05	34.0 [16.0-52.0]-36.7 [21.0-52.4]
2017	Congo, Democratic Republic	23.8	0.36	28.5 [17.4-39.5]-52.3 [43.0-61.6]
2014	Congo, Republic	21.7	0.41	30.7 [21.2-44.2]-52.4 [47.0-58.4]
2016	Cote d'Ivoire	30.1	0.36	51.7 [41.8-63.7]-81.8 [73.2-91.2]
2014	Dominican Republic	1.3	0.04	25.9 [16.5-35.4]-27.2 [21.6-32.9]
2014	Egypt, Arab Republic	18.7	0.43	17.6 [13.3-21.8]-36.2 [30.2-42.3]
2014	El Salvador	13.6	0.85	10.4 [3.6-17.2]-24.0 [14.9-33.1]
2014	Eswatini	46.7	0.66	34.3 [20.6-48.1]-81.1 [60.7-101.4]
2016	Ethiopia	7.6	0.09	54.0 [39.8-68.3]-61.6 [47.3-76.0]
2012	Gabon	2.3	0.04	40.3 [17.2-63.4]-42.6 [35.6-49.7]
2018	Gambia, The	16.9	0.42	31.7 [22.3-41.2]-48.7 [41.0-56.3]
2017	Ghana	1.4	0.03	37.7 [25.2-50.2]-39.1 [29.3-48.9]
2014	Guatemala	23.7	0.54	17.4 [12.1-22.8]-41.1 [34.7-47.6]
2018	Guinea	43.1	0.55	34.3 [24.2-44.5]-77.4 [66.3-88.5]
2018	Guinea-Bissau	8.4	0.17	43.7 [30.6-56.8]-35.2 [26.6-43.9]
2014	Guyana	7.1	0.21	26.7 [11.0-42.4]-33.8 [21.4-46.2]
2016	Haiti	14.8	0.20	48.1 [34.1-62.0]-62.8 [50.4-75.3]
2011	Honduras	12.4	0.49	17.6 [11.1-24.1]-30.0 [25.3-34.7]
2015	India	36.7	0.65	21.2 [19.7-22.6]-57.9 [56.6-59.2]
2017	Indonesia	19.6	0.52	19.9 [15.9-24.0]-39.6 [34.7-44.4]
2018	Iraq	10.7	0.41	13.7 [9.6-17.7]-24.4 [20.8-28.0]
2017	Jordan	2.5	0.12	15.3 [5.9-24.8]-17.9 [11.5-24.3]
2014	Kenya	1.3	0.02	38.4 [29.8-46.9]-39.6 [35.1-44.2]
2018	Kiribati	12.7	0.32	34.3 [18.4-50.3]-47.0 [33.9-60.1]
2018	Kyrgyz Republic	9.3	0.28	9.9 [5.6-14.3]-19.2 [9.3-29.1]
2017	Lao PDR	42.4	0.73	17.5 [11.7-23.2]-59.8 [53.7-66.0]
2018	Lesotho	0.9	0.01	63.9 [36.9-91.0]-64.9 [49.5-80.2]
2013	Liberia	17.2	0.22	62.2 [45.8-78.6]-79.4 [68.5-90.3]
2018	Madagascar	15.1	0.24	30.1 [22.0-38.2]-45.2 [39.3-51.1]
2015	Malawi	3.2	0.04	43.5 [34.9-52.1]-46.7 [40.7-52.6]
2016	Maldives	9.5	0.46	20.6 [2.8-38.4]-11.1 [5.9-16.4]
2018	Mali	40.7	0.45	36.8 [29.3-44.3]-77.5 [66.3-88.7]
2015	Mauritania	14.8	0.31	32.8 [25.7-41.9]-47.6 [41.7-54.3]
2012	Moldova	17.3	0.95	12.5 [6.3-24.8]-29.8 [16.1-54.7]
2018	Mongolia	13.4	0.71	10.3 [1.7-18.9]-23.7 [17.7-29.7]
2015	Mozambique	12.9	0.14	32.1 [19.1-45.1]-19.2 [10.4-28.0]
2015	Myanmar	55.4	1.05	22.5 [11.3-33.7]-77.9 [64.0-91.8]
2013	Namibia	28.2	0.67	22.4 [10.9-34.0]-50.7 [40.2-61.2]
2019	Nepal	17.5	0.32	15.1 [7.3-23.0]-32.6 [24.8-40.4]
2012	Niger	11.3	0.12	52.4 [42.6-62.3]-63.8 [54.1-73.4]
2018	Nigeria	38.5	0.49	39.6 [34.1-45.2]-78.1 [71.6-84.6]
2018	North Macedonia	10.8	0.71	16.7 [0.0-36.8]-27.4 [7.0-47.8]
2017	Pakistan	23.7	0.33	52.5 [39.6-65.5]-76.3 [66.0-86.5]
2016	Papua New Guinea	19.6	0.53	27.4 [19.0-35.7]-47.0 [32.3-61.7]
2016	Paraguay	21.5	1.28	4.5 [0.2-8.8]-26.0 [16.2-35.7]
2018	Peru	11.3	0.50	10.6 [3.5-17.7]-21.9 [17.8-26.0]
2017	Philippines	21.6	0.78	9.1 [3.9-14.4]-30.7 [24.8-36.7]
2014	Rwanda	24.9	0.31	25.0 [18.9-31.1]-50.0 [41.6-58.3]
2019	Sao Tome and Principe	1.8	0.06	18.3 [6.9-29.7]-20.1 [10.3-30.0]
2017	Senegal	19.1	0.39	27.3 [18.9-35.7]-46.3 [40.8-51.9]
2017	Sierra Leone	16.8	0.18	73.5 [62.4-84.6]-56.7 [50.5-63.0]
2016	South Africa	14.5	0.35	39.8 [9.1-70.5]-54.2 [40.4-68.1]
2010	South Sudan	13.4	0.2	78.6 [67.5-89.8]-65.2 [55.9-74.6]
2014	Sudan	25.2	0.47	35.2 [28.7-41.7]-60.4 [54.1-66.8]
2018	Suriname	7.7	0.44	9.9 [0.4-19.3]-17.6 [10.7-24.4]
2017	Tajikistan	21.5	0.66	18.0 [12.0-24.0]-39.5 [30.1-48.9]
2015	Tanzania	13.9	0.18	58.6 [45.7-71.6]-44.8 [36.0-53.5]
2016	Timor-Leste	15.5	0.36	20.2 [12.1-28.3]-35.7 [27.1-44.2]
2013	Togo	29.8	0.46	31.6 [22.5-40.7]-61.4 [52.3-70.6]
2019	Tonga	14.6	1.57	1.1 [0.0-3.2]-15.7 [3.5-27.9]
2018	Tunisia	16.1	1.12	4.8 [0.7-8.8]-20.9 [14.0-27.8]
2013	Turkiye	14.9	0.36	7.8 [0.0-16.3]-22.6 [15.3-30.0]
2019	Turkmenistan	10.6	0.37	21.3 [12.4-30.3]-32.0 [22.0-42.0]
2016	Uganda	17.1	0.23	39.2 [32.3-46.1]-56.3 [49.1-63.6]
2013	Vietnam	20.6	0.88	8.4 [3.1-13.7]-29.1 [18.9-39.2]
2013	Yemen, Republic	20.3	0.36	33.0 [25.8-40.2]-53.3 [45.7-60.9]
2018	Zambia	2.8	0.04	41.5 [30.2-52.8]-44.3 [37.0-51.6]
2019	Zimbabwe	23.9	0.43	38.8 [28.6-49.0]-62.7 [49.8-75.6]

**Table 2.** Slope Index of Inequality (SII) and Relative Index of Inequality (RII) (Higher = More Unequal) for Under-Five Mortality Rate (U5MR; Deaths per 1,000 Live Births) for the Most Recent Year for Each Country Included.

Year	Country	SII	RII	U5MR for Wealth Quintile 5 (richest) – Quintile 1 (poorest) with 95% CIs
	*Average*	*30*.*7*	*0*.*5*	*Q5: 38.0 Q1: 67.8*
	*Range*	*1.5-119.4*	*0.02-1.4*	*Q5: 0.4 −113.7 Q1: 14.3-174.8*
2015	Afghanistan	40.6	0.66	39.9 [32.4-47.4]-80.5 [71.3-89.7]
2017	Albania	13.9	1.05	0.4 [0.0-1.1]-14.3 [5.6-23.1]
2018	Algeria	9	0.41	13.4 [7.3-19.6]-22.4 [17.9-27.0]
2015	Angola	63.3	0.83	38.8 [25.4-52.2]-102.2 [88.1-116.3]
2015	Armenia	10.1	0.36	4.5 [0.0-10.1]-14.6 [5.8-23.5]
2019	Bangladesh	22.3	0.26	28.2 [24.0-32.5]-50.6 [45.6-55.5]
2015	Belize	17.7	1.13	3.2 [0.0-7.2]-20.9 [9.5-32.4]
2017	Benin	47.7	0.38	60.1 [51.1-69.1]-107.8 [96.0-119.6]
2010	Burkina Faso	78.3	0.42	96.6 [84.0-109.1]-174.8 [159.9-189.8]
2016	Burundi	67.4	0.66	51.6 [40.9-62.3]-119.0 [107.5-130.5]
2014	Cambodia	57.7	0.71	18.7 [12.8-24.6]-76.4 [61.5-91.3]
2018	Cameroon	61.7	0.52	49.0 [39.7-58.3]-110.7 [95.5-125.9]
2018	Central African Republic	50	0.39	67.9 [55.6-80.2]-117.9 [100.5-135.3]
2019	Chad	13.8	0.08	91.4 [79.8-103.0]-105.2 [94.2-116.3]
2015	Colombia	20.3	0.82	6.8 [2.0-11.6]-27.2 [21.9-32.4]
2012	Comoros	11.9	0.15	40.1 [21.2-58.9]-52.0 [33.8-70.1]
2017	Congo, Democratic Republic	46.2	0.42	39.8 [26.7-52.8]-86.0 [74.2-97.8]
2014	Congo, Republic	46.7	0.56	32.1 [22.1-46.3]-78.7 [72.1-85.9]
2016	Cote d'Ivoire	48.5	0.37	72.9 [60.8-87.2]-121.4 [109.2-134.8]
2014	Dominican Republic	7.9	0.19	26.4 [17.0-35.7]-34.3 [28.1-40.5]
2014	Egypt, Arab Republic	22.9	0.43	19.2 [14.8-23.7]-42.2 [35.9-48.5]
2014	El Salvador	17.5	0.93	13.2 [5.2-21.1]-30.7 [20.7-40.7]
2014	Eswatini	51.9	0.55	50.8 [27.4-74.1]-102.6 [81.3-124.0]
2016	Ethiopia	23	0.18	66.7 [52.2-81.2]-89.7 [71.5-108.0]
2012	Gabon	25	0.33	50.3 [27.1-73.5]-75.3 [64.1-86.4]
2018	Gambia, The	37.6	0.64	39.1 [29.6-48.6]-76.6 [64.2-89.1]
2017	Ghana	14.6	0.16	48.2 [31.9-64.5]-62.8 [51.4-74.2]
2014	Guatemala	36	0.62	20.1 [14.2-25.9]-56.0 [47.8-64.3]
2018	Guinea	88.5	0.64	44.4 [33.3-55.5]-132.9 [115.5-150.4]
2018	Guinea-Bissau	1.5	0.02	58.6 [40.4-76.9]-60.2 [48.2-72.2]
2014	Guyana	8.7	0.23	30.6 [15.1-46.2]-39.4 [25.9-52.8]
2016	Haiti	34.7	0.32	58.6 [44.0-73.1]-93.3 [78.7-107.8]
2011	Honduras	18.7	0.58	20.2 [13.0-27.5]-38.9 [33.9-43.9]
2015	India	50.7	0.68	24.5 [22.8-26.2]-75.2 [73.5-76.9]
2017	Indonesia	28.6	0.58	23.9 [19.4-28.4]-52.5 [46.3-58.6]
2018	Iraq	13	0.43	16.3 [11.8-20.9]-29.3 [25.9-32.8]
2017	Jordan	3.1	0.13	16.3 [6.8-25.8]-19.4 [12.9-25.9]
2014	Kenya	10.1	0.11	46.6 [37.0-56.2]-56.7 [51.2-62.3]
2018	Kiribati	25.7	0.47	40.7 [23.8-57.6]-66.4 [50.6-82.1]
2018	Kyrgyz Republic	10.4	0.26	11.0 [6.6-15.3]-21.3 [10.9-31.8]
2017	Lao PDR	46.5	0.68	20.5 [14.6-26.4]-67.0 [61.0-73.1]
2018	Lesotho	3	0.03	80.3 [52.4-108.1]-83.3 [66.2-100.4]
2013	Liberia	30.4	0.24	99.4 [78.5-120.3]-129.8 [113.2-146.4]
2018	Madagascar	35.6	0.36	39.1 [29.2-48.9]-74.6 [64.7-84.5]
2015	Malawi	23	0.18	60.1 [50.6-69.7]-83.2 [74.8-91.5]
2016	Maldives	7.6	0.32	22.6 [4.4-40.8]-15.0 [9.1-20.9]
2018	Mali	85.3	0.5	57.3 [47.3-67.2]-142.6 [127.6-157.6]
2015	Mauritania	27.8	0.45	39.7 [32.4-48.5]-67.5 [60.3-75.6]
2012	Moldova	17.3	0.82	12.5 [6.3-24.8]-29.8 [16.1-54.7]
2018	Mongolia	15.6	0.69	14.2 [4.8-23.6]-29.8 [23.5-36.0]
2015	Mozambique	14.5	0.11	47.6 [28.8-66.4]-33.1 [20.3-46.0]
2015	Myanmar	72.9	1.13	25.9 [14.3-37.6]-98.8 [82.7-114.9]
2013	Namibia	36.1	0.59	30.7 [17.0-44.3]-66.8 [54.6-79.0]
2019	Nepal	20.1	0.28	19.7 [9.4-29.9]-39.7 [31.1-48.3]
2012	Niger	30.2	0.14	113.7 [98.0-129.4]-143.9 [129.2-158.5]
2018	Nigeria	119.4	0.81	53.4 [47.2-59.5]-172.8 [160.6-184.9]
2018	North Macedonia	20	1.17	16.7 [0.0-36.8]-36.6 [4.4-68.8]
2017	Pakistan	43.6	0.5	56.3 [42.2-70.5]-99.9 [86.7-113.1]
2016	Papua New Guinea	33.2	0.65	35.9 [26.3-45.6]-69.2 [51.6-86.7]
2016	Paraguay	25	1.36	4.5 [0.2-8.8]-29.5 [19.6-39.5]
2018	Peru	12.2	0.42	15.3 [4.3-26.2]-27.5 [22.9-32.0]
2017	Philippines	30.8	0.78	11.2 [5.5-16.9]-42.0 [34.5-49.5]
2014	Rwanda	44.4	0.32	40.0 [31.6-48.4]-84.4 [73.4-95.4]
2019	Sao Tome and Principe	8.9	0.21	18.8 [7.3-30.4]-27.7 [15.6-39.9]
2017	Senegal	45.8	0.56	30.0 [21.1-38.8]-75.7 [67.5-83.9]
2017	Sierra Leone	6.6	0.04	101.0 [88.5-113.4]-94.4 [87.0-101.8]
2016	South Africa	25.8	0.5	41.3 [10.5-72.1]-67.2 [52.0-82.3]
2010	South Sudan	10.8	0.11	105.0 [93.3-116.8]-94.2 [83.7-104.8]
2014	Sudan	38	0.51	42.6 [34.5-50.7]-80.7 [73.6-87.8]
2018	Suriname	10	0.51	10.5 [0.6-20.4]-20.5 [13.0-27.9]
2017	Tajikistan	26.2	0.65	20.4 [14.3-26.5]-46.7 [36.2-57.1]
2015	Tanzania	4.9	0.04	72.8 [57.5-88.1]-77.6 [66.0-89.2]
2016	Timor-Leste	29.7	0.49	25.1 [15.8-34.5]-54.8 [45.0-64.6]
2013	Togo	74.2	0.65	46.3 [34.9-57.6]-120.5 [106.6-134.3]
2019	Tonga	14.6	1.44	1.1 [0.0-3.2]-15.7 [3.5-27.9]
2018	Tunisia	20.5	1.18	5.4 [1.0-9.8]-25.9 [17.5-34.3]
2013	Turkiye	19.8	0.4	8.1 [0.0-16.6]-27.9 [20.2-35.6]
2019	Turkmenistan	16.5	0.49	23.1 [13.4-32.7]-39.5 [28.6-50.5]
2016	Uganda	35.7	0.28	52.6 [44.0-61.3]-88.4 [79.8-97.0]
2013	Vietnam	26.3	0.85	9.8 [4.4-15.3]-36.1 [24.4-47.8]
2013	Yemen, Republic	31.3	0.46	37.8 [30.2-45.4]-69.1 [60.7-77.6]
2018	Zambia	9.1	0.07	57.4 [44.6-70.2]-66.6 [57.7-75.4]
2019	Zimbabwe	40.1	0.51	51.0 [37.6-64.4]-91.1 [68.8-113.3]

As suggested in Tables 1 and 2, data did not conform to the expected socioeconomic gradient in mortality for the Maldives, Mozambique, Sierre Leone, or South Sudan (and Guinea-Bissau and Tanzania for infant mortality), with mortality rates varying in an unclear pattern across wealth quintiles for these countries on these indicators.

The results of the mixed effect regression analyses are shown in Table 3. A country's GDP per capita predicted absolute inequities in both infant and under-five mortality, with a medium effect size (*f*^2^ = 0.26 for infant mortality; *f*^2^ = 0.33 for under-five mortality).^49^ GDP per capita predicted relative inequities in infant mortality and under-five mortality with small effect sizes (*f*^2^ ≥ 0.02).^49^ The higher a country's income, the lower its absolute inequities in under-five mortality were, but the higher its relative indexes of inequalities for infant and under-five mortality were on average.

**Table 3.** Mixed Effect Regression Analyses for the Relationship Between Gross Domestic Product (GDP) per Capita and Inequities in Infant and Under-Five Mortality.

	N	Coefficient (95% CI)	*P*-value	R^2^	*f* ^2^
SII (Absolute inequity)					
Infant mortality	83	−10.26 (−13.18-−7.34)	<.001	0.53	0.26
Under-five mortality	83	−24.71 (−29.61-−19.81)	<.001	0.76	0.33
RII (Relative inequity)					
Infant mortality	83	0.11 (0.05-−0.17)	<.001	0.48	0.08
Under-five mortality	83	0.11 (0.05-0.16)	<.001	0.60	0.07

Note: Log transformation of GDP; R^2^ represents the variance explained by the entire model, including both fixed and random effects; *f*^2^ is a measure of effect size.

**Figure 1.**
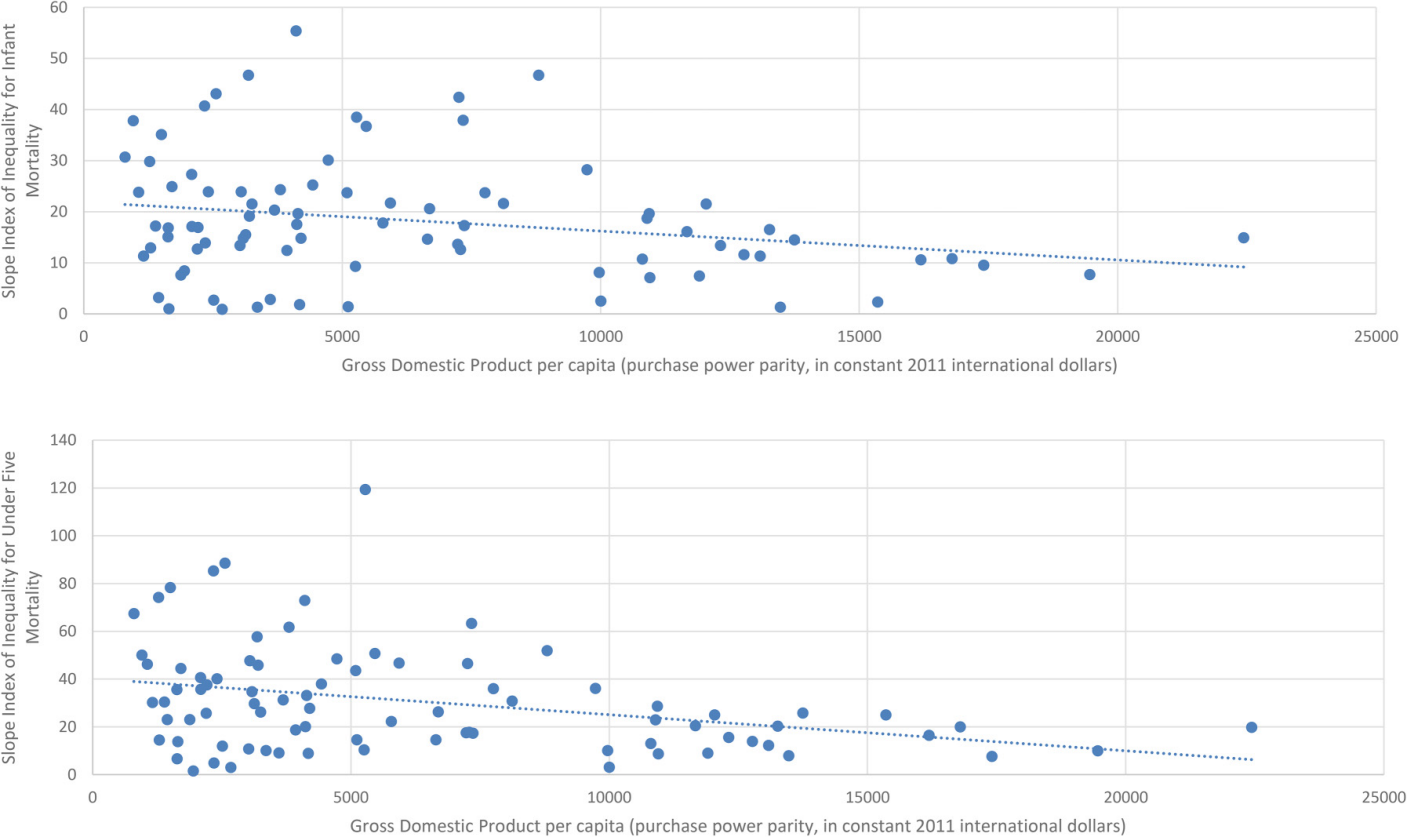
Scatter Diagram of the Relationship between Gross Domestic Product Per Capita and the Slope Index of Inequality for Infant Mortality (Top) and Under-five Mortality (Bottom) for the Most Recent Year Available.

**Figure 2.**
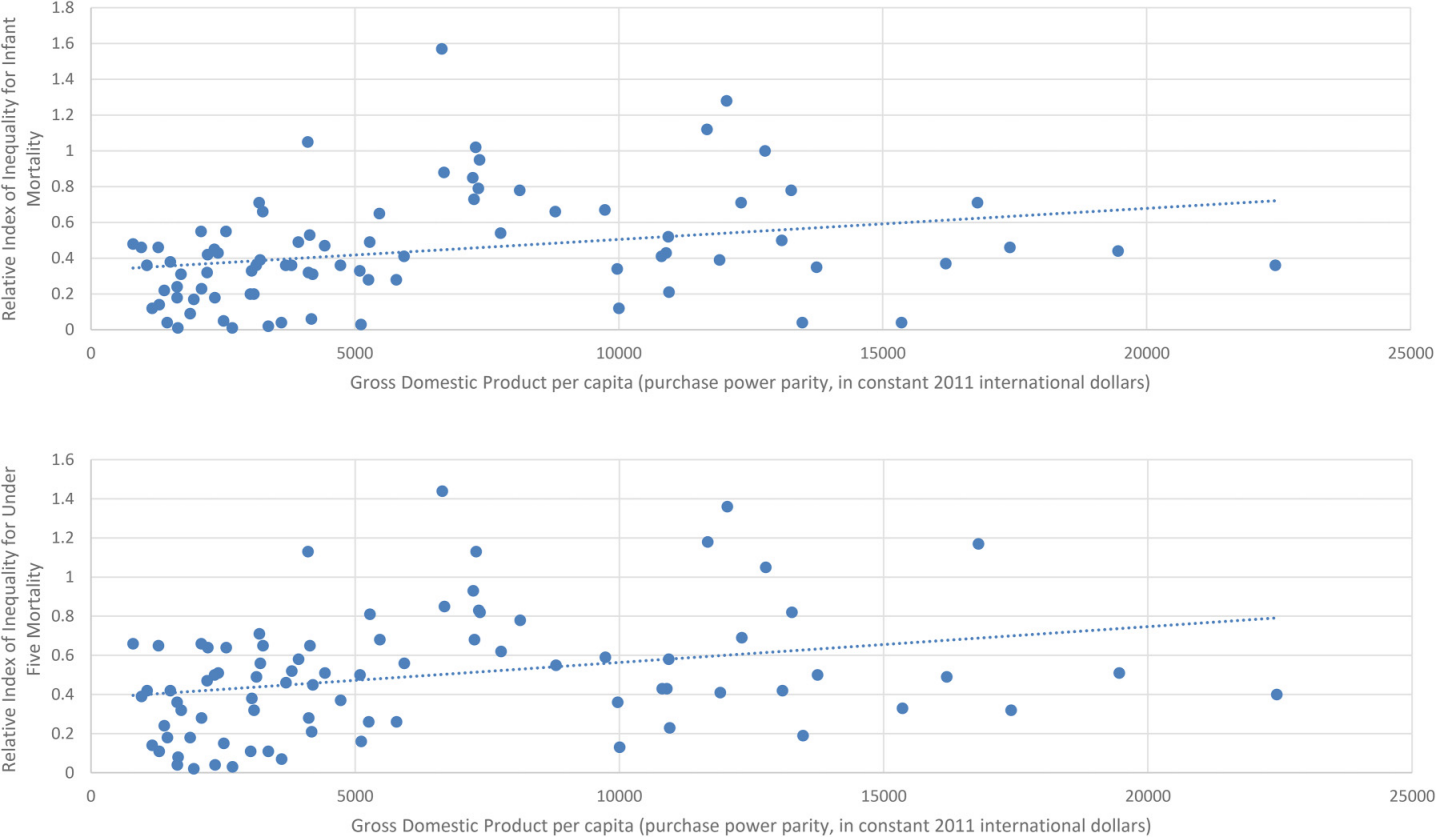
Scatter Diagram of the Relationship between Gross Domestic Product Per Capita and the Relative Index of Inequality for Infant Mortality (Top) and Under-five Mortality (Bottom) for the Most Recent Year Available.

Scatterplots for SIIs for the most recent year available for each country are shown in Figure 1, and the scatterplot for RIIs for the most recent year available for each country are shown in Figure 2.

## Discussion

Our study yielded three key findings. First, based on our calculations of SIIs of 83 LMICs, we found that social gradients in infant and under-five mortality were evident in most countries, and in many cases, inequities were very large. Such extensive within-country health inequities represent a failure to achieve health for all. These inequities highlight the shortcoming of relying on average health measures to compare countries on population health outcomes, as these are liable to hide these critical health inequities. For the few countries where the mortality rates did not follow a social gradient of inequities, mortality rates varied with no clear pattern across quintiles, suggesting lower quality data rather than fewer inequities. That these countries (Maldives, Mozambique, Sierre Leone, South Sudan, and Guinea-Bissau and Tanzania for infant mortality) do not have a social gradient seems highly unlikely given what we know of how health gets distributed in a population.^25^ Since these nations with governance concerns, political instability, economic inequities, and/or a history of civil war, the findings may likely be the result of problems with data collection rather than the lack of a social gradient.

Second, we found that among LMICs, a higher GDP was associated with a significant decrease in absolute inequalities in both infant and under-five mortalities. This finding shows that as well as the strong evidence for a higher GDP being associated with greater overall life expectancy, with particularly positive gains amongst LMICs,^5^ a higher GDP is also likely to reduce absolute inequalities in child mortality.

Third, we found a positive association between GDP and relative inequities in infant and under-five mortality. This association may be an artifact of lower overall infant and under-five mortality in countries with higher income; as mortality levels drop, relative inequities mathematically tend to increase.^50^ Conversely, absolute inequities tend to decrease,^48^ which may contribute to the decrease in absolute inequities we found. Alternatively, our findings may raise concerns about how the benefits of higher GDPs get distributed among the population. If increasing GDP is increasing relative inequities, then it is likely that the benefits of a higher GDP are not being distributed in a way that can lead to a flattening of the socioeconomic gradient in health. Our findings add to calls to ensure equity in the distribution of national wealth and income to ensure everyone has the opportunity for good health.^3,51,52^

Reductions in child mortality have been achieved through action on the social determinants of health including household wealth, improvements in water and sanitation, immunizations, and education.^6,53^ Technologies in the home such as fuel used for cooking and refrigeration have also affected child mortality rates and may improve with country and household wealth.^6,54^ Thus, the association we found between absolute inequalities in infant and under-five mortality and GDP suggests that increasing national wealth may have led to more equitable distribution of these determinants. The fact that the reduction in inequalities in infant and under-five mortality as GDP increased were not large (*f*^2^ = 0.26 and 0.33) and were accompanied by a small increase in relative inequities in infant and under-five mortality suggests that there is scope to improve the equitable distribution of increasing national wealth. The fact that the data shows these inequities continue confirms that inequities in infant and under-five mortality remain crucial public health issues at all levels of development among LMICs.

One priority for future research and public health action is to address the lack of available comparable data for high-income countries. We were only able to find comparable infant mortality data by socioeconomic quintile for four high-income countries. We could not find under-five mortality by socioeconomic quintile for any high-income country. This problem represents a significant gap in our knowledge and makes it impossible to assess progress in reducing infant or under-five mortality inequities in most high-income countries. It also means we lacked global measures of health inequities by socioeconomic status by which to compare all countries—a resource that would allow much investigation of amenable drivers to reduce health inequities through public policy and other strategies. Much could be learned about successful policy settings and approaches from countries that are minimizing health inequities, and about deleterious policies and drivers from countries that have higher health inequities.

### Limitations of This Study

The study was limited by the available data, and comprehensive comparative analysis of health inequities between countries would require stronger comparable health inequities data than are currently available. The shortcomings of available data are a key finding in our study. The Health Equity Monitor is a valuable resource, but not sufficient to allow full exploration of health inequities within countries globally.

In addition, the time points for which the Healthy Equity Monitor data were available differed between countries (1996-2019, though the nine countries with data older than 2010 were excluded from analyses), reducing comparability, and confidence intervals around the data points were very large. Reducing child mortality has been a goal of the Millennium Development Goals, the Sustainable Development Goals, and the U.N. Development Program.^6,34^ One analysis found that child mortality, and socioeconomic inequities in child mortality, have decreased in LMICs since the 1990s, while relative socioeconomic inequities in child mortality have remained stable.^34^ Improvements in health care systems over time and the progress towards universal health coverage are also likely to improve child mortality and inequities in child mortality.^55^

Lastly, these data points predate the COVID-19 pandemic, which has highlighted and exacerbated the health inequities that exist in all countries, along gender, socioeconomic, and ethnicity lines, and this fact has brought renewed urgency to the task of understanding the causes of inequities.^21–24^

## Conclusions

Unlike the strong association between a country's income and their overall level of health, in our study of LMICs we found a higher GDP was associated with a medium reduction in absolute socioeconomic inequities in both infant and under-five mortality, and a small increase in the relative inequities in both mortality categories. While the decrease in absolute inequities is positive, considerable inequities in infant and under-five mortality remain, and the finding that relative inequities in mortality increased with GDP indicate that there is scope to improve the extent to which benefits of a higher GDP are being used to develop policies designed to flatten socioeconomic gradients in health. Understanding what countries’ contextual factors beyond GDP drive absolute and relative health inequities is a critical population health problem and should be theorized and empirically examined further.^10^
